# *Enterococcus durans* Cardiac Implantable Electronic Device Lead Infection and Review of *Enterococcus durans* Endocarditis Cases

**DOI:** 10.3390/medicina58020307

**Published:** 2022-02-17

**Authors:** Milan Radovanovic, Djordje Jevtic, Michel K. Barsoum, Janki Patel, Igor Dumic

**Affiliations:** 1 Mayo Clinic Alix School of Medicine, Rochester, MN 55905, USA; barsoum.michel@mayo.edu (M.K.B.); dumic.igor@mayo.edu (I.D.); 2 Department of Hospital Medicine, Mayo Clinic Health System, Eau Claire, WI 54703, USA; 3 School of Medicine, University of Belgrade, 11000 Belgrade, Serbia; djordje965@gmail.com; 4 Department of Cardiology, Mayo Clinic Health System, Eau Claire, WI 54703, USA; 5 Department of Infectious Disease, Mayo Clinic Health System, Eau Claire, WI 54703, USA; janki.n.patel78@gmail.com

**Keywords:** *Enterococcus durans*, cardiac implantable electronic device (CIED) lead, endocarditis, bacteremia

## Abstract

*Introduction:* Cardiac implantable electronic device (CIED) infections present a growing problem in medicine due to a significant increase in the number of implanted devices and the age of the recipient population. *Enterococcus* spp. are Gram-positive, facultative anaerobic, lactic acid bacteria; they are relatively common pathogens in humans, but uncommon as the cause of CIED lead infections. Only eight cases of *Enterococcus durans* endocarditis have been reported in the literature thus far; however, there are no reported cases of *Enterococcus durans* CIED lead infection. *Case presentation***:** A 58-year-old gentleman with a previously implanted St. Jude Medical single-chamber implantable cardioverter–defibrillator (ICD) due to tachy/brady arrhythmias presented with nonspecific constitutional symptoms (i.e., low-grade fevers, chills, fatigue), and was found to have innumerable bilateral pulmonary nodules via computed tomography angiography of the chest. Many of these pulmonary nodules were cavitated and highly concerning for septic pulmonary emboli and infarcts. Within 24 h from presentation, blood cultures in all four culture bottles grew ampicillin- and vancomycin-susceptible *Enterococcus durans*. Transthoracic echocardiogram confirmed vegetations on the ICD lead in the right ventricle. The patient underwent laser extraction of the ICD lead with generator removal and recovered completely after a 6-week intravenous antibiotic course. *Conclusion***:** To our knowledge, this is the first report of CIED lead infection caused by *Enterococcus durans*. In this case, management with antibiotics along with ICD lead extraction led to complete recovery. Clinicians should be aware of this rare but potentially devastating infection in patients with native and artificial valves, but also in those with CIEDs.

## 1. Introduction

*Enterococcus* spp. (previously classified as a group-D *Streptococcus*) are Gram-positive, catalase- and oxidase-negative, facultative anaerobic, lactic acid bacteria that are part of the *Enterococcaceae* family, *Lactobacillales* order, and *Bacilli* class [1]. Most species of this large family live as commensals in the gastrointestinal tracts (GITs) of various organisms, including humans, animals, and insects. Although a part of the microbiome, some *Enterococcus* species can cause a variety of infections in their hosts, including severe infections such as infective endocarditis (IE) [1]. Most common enterococcal infections in humans are caused by *Enterococcus faecalis* *(E. faecalis)* (90–95%) and *Enterococcus faecium (E. faecium)* (5–10%) [1]. *Enterococcus durans (E. durans)*, previously known as *Streptococcus durans*, is a rare member of non-faecalis/non-faecium enterococcal species, and is usually found in the intestines of animals [2]. *E. durans* infrequently causes infection in humans, and is an exceedingly rare cause of IE [2]. We report a case of *E. durans* cardiac implantable electronic device (CIED) infection, which has not been reported in the literature thus far. CIED infections can sometimes progress to CIED-related infective endocarditis (CIED-IE) when valvular or lead vegetations are present, or when the Duke criteria are met with positive blood and/or lead tip cultures [3].

## 2. Case Presentation

A 58-year-old Caucasian gentleman presented to the emergency department with complaints of a 1-day-long pain in the left shoulder, upper chest, and back. Additionally, he reported a 3-week history of fatigue, malaise, nighttime chills, and rigors. His cardiac history was significant for non-ischemic cardiomyopathy and chronic heart failure with reduced ejection fraction (New York Heart Association (NYHA) class II, stage C). He had a St. Jude Medical single-chamber implantable cardioverter–defibrillator (ICD) placed in the right ventricle (RV) 2 years prior for tachy/brady arrhythmias (interrogation showed ventricular tachycardia with 2–3 ICD discharges since placement, the most recent occurring 1 year prior). His other cardiac history was notable for mild non-obstructive coronary artery disease noted upon cardiac catheterization carried out 1 year prior, permanent atrial fibrillation (AF)—for which he was anticoagulated with warfarin—and controlled essential hypertension. The patient worked in the information technology (IT) industry; he denied tobacco product use and consumed alcohol only socially.

**Figure 1:**
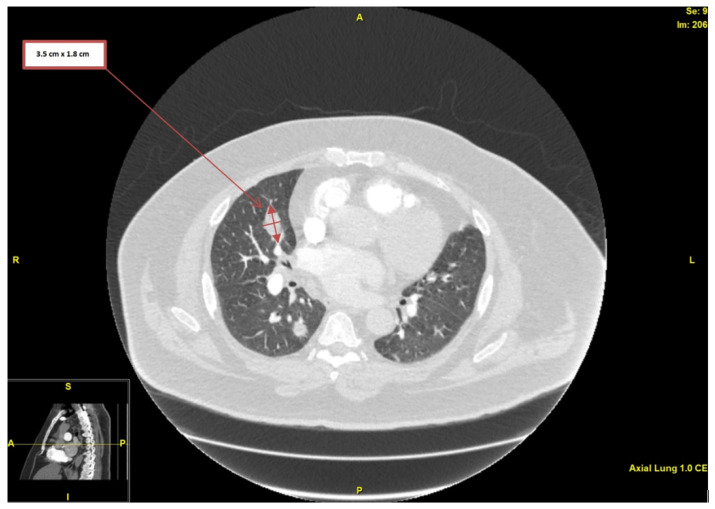
Computed tomography angiography of the chest, showing nodularity with associated cavitation (largest measuring 3.5 × 1.8 cm in diameter).

Upon physical examination, the patient was afebrile, and his vital signs were within normal limits, with only mild tachycardia of around 105 beats per minute. There were no loud heart murmurs or any of the endocarditis stigmata (i.e., splinter hemorrhages, Janeway lesions, or Osler nodes). His initial laboratory findings demonstrated elevated erythrocyte sedimentation rate (ESR) and elevated C-reactive protein (CRP)—120 mm/h and 49.6 mg/L, respectively—but his white blood cell (WBC) count was within normal limits. His creatinine level was 0.85 mg/dL, with an estimated glomerular filtration rate (eGFR) > 90 mL/min/1.73 m^2^. His international normalized ratio (INR) was 1.5, and his D-dimer was elevated at 1.24 mcg/mL (reference value: below 0.58 mcg/mL adjusted for age). Electrocardiogram (ECG) showed AF with rapid ventricular response, but without any ischemic changes. Computed tomography angiography (CTA) of the chest was performed primarily to rule out pulmonary embolism (PE), but demonstrated innumerable bilateral pulmonary nodules, many being cavitated (the largest one measuring 3.5 × 1.8 cm in diameter) and highly concerning for septic pulmonary emboli and infarcts (Figure 1). 

**Figure 2:**
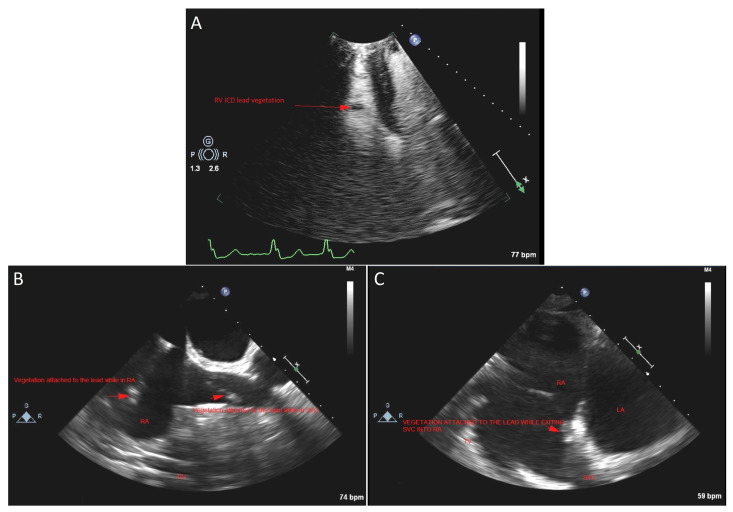
(**A**) Transthoracic echocardiogram (TTE) view that shows right ventricle (RV) ICD lead vegetation. (**B**) Transesophageal echocardiogram (TEE) view that shows vegetation attached to the ICD lead in the superior vena cava (SVC), but also vegetation attached to the lead while in the right atrium (RA). (**C**) TEE view that shows a small mass attached to the ICD lead while exiting the SVC into the RA, suspicious for vegetation. LA: left atrium.

The patient then underwent transthoracic echocardiogram (TTE), which revealed large ICD lead vegetations in the RV (Figure 2A). Moderate aortic valve regurgitation was noted, but without any apparent valve vegetations. Due to mild symptoms, the absence of fever, and hemodynamic stability, it was decided to defer antibiotic initiation until further workup was done. After 12 h, all four blood culture samples (two aerobic, two anaerobic) were positive for ampicillin-susceptible, vancomycin-susceptible *E. durans*, with minimum inhibitory concentrations (MICs) of ≤ 2 mcg/mL and ≤ 0.5 mcg/mL, respectively (BD BACTEC Plus Aerobic and Lytic/10 Anaerobic F Culture; identification was carried out via Bruker MALDI-TOF mass spectrometry, and the antimicrobial susceptibility test was conducted using a bioMérieux VITEK 2 XL instrument, Mayo Clinic Laboratories). Consequently, therapy for enterococcal endocarditis was initiated with 2 g of intravenous (IV) ampicillin every 4 h and 2 g of ceftriaxone every 12 h. Blood cultures were carried out daily for the following 5 days, and they were repeatedly positive for the first 2 days, but afterwards remained negative. Five days after TTE was done, transesophageal echocardiogram (TEE) revealed a small mass attached to the ICD lead in the superior vena cava (SVC) (Figure 2B) while exiting into the right atrium (RA) (Figure 2C), which was suspicious for vegetation; however, the RV portion of the lead did not reveal any vegetation previously seen on TTE. 

**Figure 3:**
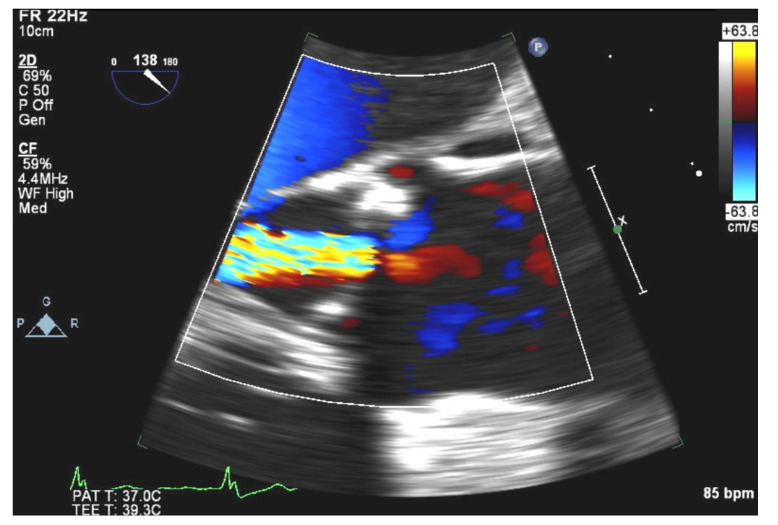
TEE view showing sclerotic aortic valve with moderate aortic valve regurgitation.

This raised the suspicion that previously noted vegetations had likely embolized. TEE confirmed sclerotic aortic valve with moderate aortic valve regurgitation indicative of unrelated valve disease (Figure 3), but failed to demonstrate any valvular vegetations.

**Figure 4:**
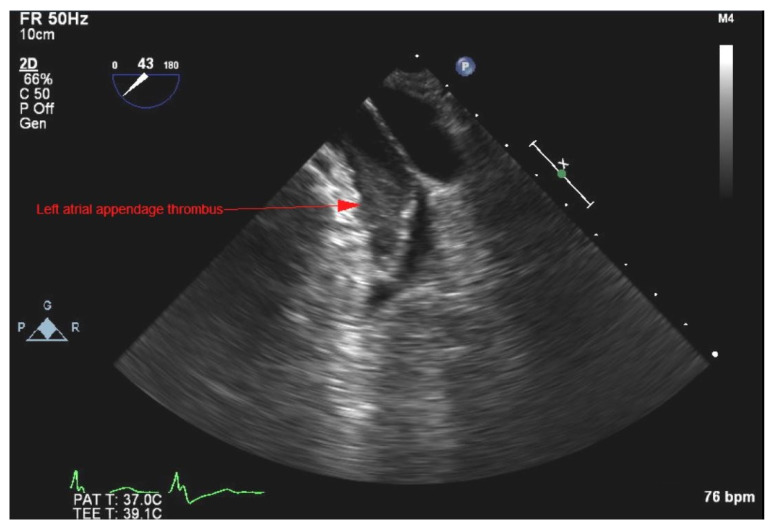
TEE view showing left atrium (LA) appendage thrombus.

There was no left-to-right shunting noted. Left atrium appendage (LAA) thrombus (Figure 4) was noted on TEE, attributed to the sub-therapeutic INR, and was deemed unrelated to vegetation as there was no interatrial shunt. 

The patient was started on a heparin drip with intention to bridge until therapeutic INR was achieved. The following day, the RV ICD lead was extracted via laser with generator removal. Operative cultures from the tissue capsule and ICD lead tip were sent for inspection, but they remained without bacterial growth. The patient was continued on the same IV antibiotic therapy, leading to improvement of inflammatory markers (CRP normalized within a week of antibiotic initiation). The patient was discharged after 2 weeks of inpatient care to a nursing facility, where he completed a 6-week antibiotic course. Apart from pulmonary nodular cavitations discovered on the CTA of the chest, the patient did not have any other embolic phenomena. Two months after completion of antimicrobial therapy, the patient continued to do well, without any signs or symptoms of recurrent disease. Follow-up TTE demonstrated no lead or valvular vegetations and, given the patient’s stable overall condition without valvular dysfunction, TEE was not repeated.

## 3. Discussion

Use of CIEDs has become increasingly prevalent in patients due to advances in cardiovascular research and technological development. These devices include pacemakers, ICDs, and cardiac resynchronizing therapy (CRT). They are constructed out of two main parts: a generator, and 1–3 wires/leads. Any part can become infected and lead to serious and devastating complications. With the rising numbers of implanted devices and the increasing age of the recipient population, there is an increased incidence of CIED infections—approximately 3.4% in the first year after implantation [4]. Infections can occur during implantation or manipulation of lead(s) and/or the pulse generator; more often, they are caused by hematogenous spread from a distant infectious source. As bacteria form biofilms on foreign objects, surrounding tissue can become infected, leading to CIED-IE, which constitutes around 10% of reported IE cases [5]. Risk factors for CIED infection are host-related (e.g., diabetes mellitus (DM), renal disease, chronic obstructive pulmonary disease, corticosteroid use, malignancy, heart failure, and anticoagulant drug use), procedure-related (e.g., lack of antibiotic prophylaxis, revision procedures, post-operative complications), or device-related (e.g., abdominal pockets, dual-chamber systems, two or more leads) [6,7]. The most common pathogens involved in CIED infections are staphylococcal species, followed by Gram-negative pathogens, streptococci, enterococci, and anaerobic species [4,8]. *Staphylococcus aureus* is the leading cause owing to its adherence factors (e.g., clumping factor A, fibronectin-binding proteins A and B), ability to form biofilm, and microbial resistance (e.g., phenotype alterations, small-colony variants) [9]. Enterococci possess traits that, although different in structure, act in a similar way, and allow bacteria to persist on foreign objects. These include gelatinase–zinc metalloprotease (mediates binding), Esp protein (allows colonization and biofilm formation), and different aggregation substances [10]. In general, studies and case reports focus on two of the most common pathogens in the Enterococcus family: *E. faecalis* and *E. faecium*. These are the second most common cause of urinary tract infections and third most common cause of nosocomial bacteremia [10]. However, *E. durans* is an extremely rare infection in humans, responsible for only 0.1% of enterococci bacteremia cases [11]; it is an exceptionally rare cause of IE, and CIED infection has never been reported before. 

**Figure 5:**
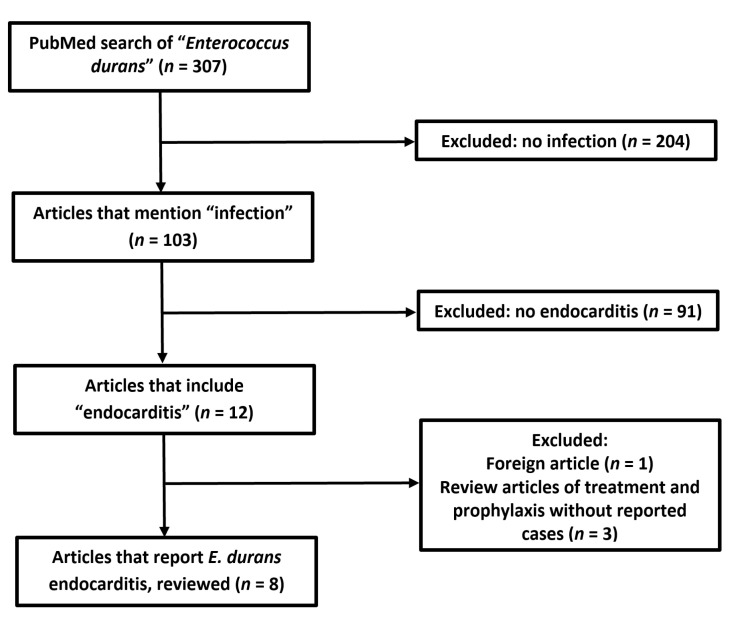
Flowchart of literature search according to Preferred Reporting Items for Systematic Reviews and Meta-Analyzes (PRISMA) guidelines.

A PubMed database search as of 8 January 2021 yielded 312 original articles that mention “*Enterococcus durans*” or “*E. durans*”; out of these, 12 articles mention “endocarditis”, and only 8 are case reports of IE caused by *E. durans* (see Figure 5 and Table 1) [2,12,13,14,15,16,17,18]. 

None of the cases mention CIED infections or CIED-IE. Review of these eight cases demonstrated that 87.5% of the patients were male or elderly (average age 65.6 years), with one case describing a female patient and one describing an infection in a 40-year-old male patient [12,17]. This younger patient had a history of transposition of great vessels, which is generally considered to increase the risk of heart infection [19]. Common comorbidities were DM (50%), hypertension (37.5%), aortic valve stenosis (37.5%), and AF (25%). Our patient was male, somewhat older, and had three of the four previously mentioned comorbidities (DM, hypertension, and AF). Further review demonstrated that native valves were affected in five patients (62.5%), followed by bioprosthetic valves in two patients (25%) and a mechanical valve in one patient (12.5%). The most commonly affected valve was aortic in four cases (50%), followed by mitral in three cases (37.5%), and there was only one case (12.5%) of tricuspid involvement in a previously mentioned male patient with non-surgically corrected transposition of the great vessels [17]. 

Compared to previously described cases, our case is unique because it represents an *E. durans* CIED lead infection—not a native or mechanical valve endocarditis which, although rare, have been reported. On presentation, our patient had vague symptoms, and our subsequent diagnostic approach was guided by discovery of the pulmonary nodular cavitations and high suspicion of septic embolism. Bacteremia was observed, with multiple sets of blood cultures testing positive for *E. durans* and ICD lead vegetations that were seen on TTE. Therefore, a definite diagnosis of CIED lead infection was made, meeting the major modified Duke criteria for IE and the Novel 2019 International CIED Infection Criteria [4]. The initial source of infection was never identified, and early initiation of the antibiotic therapy was presumed to be the reason behind the negative lead, generator, and tissue cultures that were obtained 5 days after initial presentation. Out of eight reviewed cases, only two were able to identify the source of infection: one reporting an infected brachial artery aneurysm from hemodialysis access [16], and the other reporting a colonic ulcer that served as a portal entry from the GIT [2]. 

Patients with non-faecalis, non-faecium enterococcal infection are more likely to be immunocompromised, with deficient neutrophil recruitment and impaired phagocytic activity. This can lead to mucosal defects in the GIT, bacterial translocation, and the spread of pathogens via the portal vein [2,20]. Zala et al. also concluded that alcohol consumption can increase the risk of *E. durans* sepsis [14]. Our patient was a social drinker without heavy alcohol use. He had common comorbidities for his age group (i.e., non-ischemic cardiomyopathy, chronic heart failure with reduced ejection fraction, hypertension, DM, hyperlipidemia, obesity), which can impair the immune system’s activity. Furthermore, treatment with warfarin is yet another risk factor for CIED infections [21]. More importantly, the implantation of foreign material (i.e., CIED lead) significantly increases the risk of infection [4,8]. We hypothesize that, after entry into the blood stream, there was hematogenous spread to the heart, leading to biofilm formation on the foreign object (ICD lead) that later embolized to the lungs. These emboli led to vasculature occlusion, infarction of the lungs, and emergence of pleuritic pain, which was one of the major symptoms with which the patient presented. It is unlikely that bacteria spread directly from the periphery (i.e., deep venous thrombosis, periodontal diseases, etc.), considering the lack of symptoms and the type of pathogen in question. Five days after initiating antibiotic therapy, TEE was performed, and demonstrated LAA thrombus. TEE is considered a gold standard in diagnosing LAA thrombosis, and it is possible that previous imaging modalities failed to demonstrate this finding [22]. The most common predisposing factor for intracardiac thrombosis is AF, which our patient had. Even though our patient was on warfarin, previous studies have reported the incidence of LAA thrombus in fully anticoagulated patients to be up to 10% [23]. The fact our patient’s INR was sub-therapeutic placed him at a higher risk of developing thromboembolic events. These intracardiac thrombi can be sterile or infected. Usually, in cases of infected thrombosis, there are persistent positive blood cultures despite adequate antibiotic therapy [24]. Moreover, these patients are extremely sick and usually require surgical management [24]. Considering that our patient responded to therapy exceptionally well, with rapid culture clearance, and without any additional systemic complications, we believe that this thrombus was most likely an aseptic incidental finding associated with previously diagnosed AF in the setting of sub-therapeutic INR. 

*E. durians* and the whole *Enterococcus* genus are important pathogenic agents due to the increasing number of multidrug-resistant (MDR) strains. As commensals of the GIT, they can quickly adapt to various antibiotics, become predominant flora, and cause invasive systemic infections [25]. Both enterococcal CIED-related infections and non-CIED IE are treated similarly, with systemic antibiotic therapy. Furthermore, patients with CIED infection should undergo device removal in order to reduce mortality [3,6]. The American Heart Association (AHA) and the European Society of Cardiology (ESC) recommend treating enterococcal IE with a combination of certain β-lactam antibiotics (i.e., penicillin, ampicillin, and piperacillin) and an aminoglycoside [26,27]. This combination allows for a synergistic bactericidal effect that is not feasible when the drugs are used separately [26,27]. It is reasonable that patients with native valve endocarditis (NVE) receive 6 weeks of vancomycin–gentamicin therapy, and that patients with prosthetic valve endocarditis (PVE) receive at least 6 weeks of therapy (Class IIa; Level of Evidence B) [26,27]. Prolonged therapy is necessary because of the high bacterial densities within vegetations and the relatively slow bactericidal activity of some antibiotic classes, such as β-lactams and vancomycin [26,27]. In our patient, we used a combination of two β-lactam antibiotics due to the synergistic effect of the combination, as single agent therapy may inhibit enterococci, but not necessarily kill enterococci. The dual regimen has been typically utilized in β-lactam susceptible and/or aminoglycoside resistant, *E. faecalis* endocarditis. Given, the blood culture susceptibility found pan-sensitive *E. durans,* and additionally the combination therapy avoids nephrotoxicity related to the aminoglycoside use, decision was made to treat with dual β-lactam therapy.

The most common complications of CIED infection are bacteremia with systemic complications and IE. Furthermore, IE can cause devastating intra- or extracardiac complications. Common intracardiac complications include perivalvular abscess with heart block, myocardial abscess, and chronic dysfunction of the valves with congestive heart failure [26]. The most common extracardiac complications are sepsis and/or septic embolization, with dysfunction of the organs involved [26]. Sometimes, these complications can be the initial manifestation and the reason for seeking medical help. Review of the eight *E. durans* IE cases found that complications were present in five patients (62.5%); these included myocardial infarction, aortic sinus abscess, and septic embolization to the brain, spleen, and the extremities [2,12,13,14,16]. Septic pulmonary embolization is also unique to our patient compared to systemic complications in other cases, but this is due to the right heart localization of the ICD lead compared to the left heart valves affected in other cases.

The mortality rates of CIED infection and IE are similar, and can be quite high, depending on factors such as the type of pathogen, the development of complications, and timely use of therapy. In-hospital mortality from CIED infection ranges from 3.7% to 11.3% [6], and increases up to 66% with the development of endocarditis without device removal [3]; if the device is removed, this rate can decrease to around 18% [3]. In cases of enterococcal IE, one study demonstrated a mortality rate of 11%, which was lower than but did not differ significantly from the non-enterococcal IE mortality rate of 16% [28]. Our review of *E. durans* IE cases demonstrated mortality to be as high as 37.5%, but this was based on a small sample size, and publication bias might play a significant role.

## 4. Conclusions

We describe a case of *E. durans* CIED lead infection, where the initial symptom was pleuritic chest pain from septic pulmonary emboli. The patient was successfully treated with lead extraction and 6 weeks of IV antimicrobial therapy with ampicillin and ceftriaxone. By reporting this case, we want to raise awareness of this rare and potentially fatal infection in patients with CIEDs. Early recognition and timely initiation of antimicrobial therapy are crucial to decreasing mortality, improving outcomes, and preventing further serious complications.

## Figures and Tables

**Table 1:** Summary of published *E. durans* endocarditis case reports.

Reference	Age/Sex	Valve (Anatomy)	Native or Artificial	Preceding Symptoms (Duration)	Complication (If Present)	Treatment (Abx Only or Abx + Surgery)	Length of Treatment	Outcome
**Al Shehri et al. (2020) [12]**	56 F	Mitral	Mechanical	12 weeks	Splenic infarcts	Abx only	6 weeks	Exitus
**Sunbul et al. (2018) [13]**	71 M	Mitral, anterior leaflet	Native	2 weeks	Septic emboli to brain and legs	Abx + Surgery	69 days	Exitus
**Zala et al. (2016) [14]**	61 M	Aortic	Bioprosthetic	8 weeks	AMI, septic embolus to left ophthalmic artery, bioprosthesis dysfunction	Abx + Surgery (re-do aortic valve replacement)	18 weeks	Recovered
**Fallavollita et al. (2016) [15]**	74 M	Aortic	Bioprosthetic	8 weeks	None reported	Abx only	6 weeks	Recovered
**Kenzaka et al. (2013) [16]**	83 M	Aortic	Native	16 weeks	Right brachial artery infective aneurysm (hemodialysis access fistula) with thrombus	Abx only	6 weeks	Recovered
**Vijayayakrishnan et al. (2012) [2]**	61 M	Aortic	Native	4 weeks	Aortic sinus abscesses	Abx only	6 weeks	Exitus
**Stepanovic et al. (2004) [17]**	44 M	Tricuspid	Native	12 weeks	None reported	Abx only	7.5 weeks	Recovered
**Tripodi et al.** **(1998) [18]**	74 M	Mitral	Native	1 week	None reported	Abx only	6 weeks	Recovered

M: male patient; F: female patient; Abx: antibiotics; COPD: chronic obstructive pulmonary disease; DM type 2: diabetes mellitus type 2; CHF: congestive heart failure; A-fib: atrial fibrillation; ESRD: end-stage renal disease; ESLD: end-stage liver disease; AMI: acute myocardial infarction.
